# Supplementary material for: Desmin disorganisation: A key feature in feline hypertrophic cardiomyopathy
Source: PLoS One. 2025 Jul 14;20(7):e0327850. doi: 10.1371/journal.pone.0327850 (PMC12258562; doi:10.1371/journal.pone.0327850)
Supplement: S1 Table — (DOCX) [file pone.0327850.s001.docx]

**Table S1** Clinical data, echocardiographic parameters, and histopathology diagnosis:

| Identifier | Breed | Gender | Age (yr) | BW (kg) | BCS | | La/Ao | La minor  (mm) | IVSd  (mm) | LVFWd  (mm) | LV FS% | SAM | SEC  yes/no | LA Thrombus  yes/no | Histopathology |
| --- | --- | --- | --- | --- | --- | --- | --- | --- | --- | --- | --- | --- | --- | --- | --- |
| Control 1^‡^ | Maine coon | MN | 8.5 | 5.8 | - | | 1.5 | 15.9 | 4.6 | 4.8 | 57 | No | No | No | Normal |
| Control 2^‡^ | DSH | MN | 11.3 | - | - | | 1.4 | - | - | - | - | - | No | No | Normal |
| Control 3* | Russian Blue | FN | 17 | - | - | | 1.35 | 13.8 | 4.7 | 4.7 | 61 | No | No | No | Normal |
| Control 4^‡^ | DSH | MN | 1.6 | - | - | | - | - | - | - | - | - | - | - | Normal |
| Control 5^‡^ | Russian blue | FN | 19 | - | - | | 1.52 | 14.2 | 5 | 4.8 | 57.4 | No | No | No | Normal |
| Control 6^‡^ | DSH | F | <3 | - | 5/9 | | 1.4 | - | - | - | - | - | - | - | Normal |
| Control 7* | DSH | F | <3 | - | 5/9 | | - | - | - | - | - | - | - | - | Normal |
| Control 8^‡^ | DSH | MN | 5.8 | 4.4 | 5/9 | | 1.35 | - | - | - | - | - | - | No | Normal |
| Control 9^‡^ | DSH | F | 5.8 | - | - | | - | - | - | - | - | - | - | - | Normal |
| Control 10* | DSH | F | 1.5 | - | 5/9 | | - | - | - | - | - | - | - | - | Normal |
| Control 11^†^ | DSH | FN | 6.7 | - | - | | 1.3 | - | - | - | - | - | No | No | Normal |
| Control 12^†^ | DSH | MN | 14.5 | - | - | | 1.4 | - | - | - | - | - | - | - | Normal |
| HCM 1^‡^ | BSH | MN | 13 | 4.2 | 4/9 | | 1.56 | 17 | 6.5 | 6.8 | 65.4 | No | No | No | HCM |
| HCM 2^‡^ | DSH | FN | 8.9 | 4.1 | 5/9 | | 1.61 | 18 | 6.2 | 6.4 | - | Yes | No | No | HCM |
| HCM 3* | DSH | MN | 8.4 | 6.2 | - | | 1.82 | 20.6 | 8.5 | 6.9 | 28.5 | Yes | No | No | HCM |
| HCM 4^‡^ | Main Coon | MN | 10.9 | 5.0 | 4/9 | | 1.9 | 25.4 | 6.2 | 6.1 | 49 | No | No | No | HCM |
| HCM 5^‡^ | BSH | MN | 6.9 | - | - | | 2.28 | 26 | 7.5 | 8.4 | 61 | No | Yes | No | HCM |
| HCM 6^‡^ | Bengal | FN | 15 | 3.1 | - | | 1.63 | 17 | 7.3 | 6.5 | 40.2 | Yes | Yes | No | HCM |
| HCM 7* | DSH | MN | 7 | - | - | | 1.7 | - | - | - | - | - | No | No | HCM |
| HCM 8^‡^ | Tonkinese | FN | 9 | 4.4 | - | | 2.26 | 16.9 | 7.3 | 7.1 | 61 | No | No | No | HCM |
| HCM 9* | DSH | FN | 1.7 | 3.0 | - | | 2.5 | - | - | - | - | - | No | No | HCM |
| HCM 10* | DSH | MN | 5 | 4.2 | - | | 2.6 | - | - | - | - | - | Yes | No | HCM |
| HCM 11^†^ | DSH | MN | 12.3 | 4.1 | - | | 1.68 | 23.9 | 5.4 | 6.5 | - | No | Yes | No | HCM |
| HCM 12^†^ | DLH | MN | 2 | 3.8 | - | | 2.03 | - | - | - | - | - | Yes | Yes | HCM |
| HCM 13^†^ | Ragdoll | FN | 5.4 | 4.4 | - | | 2.27 | 23.2 | 7 | 10.8 | 18 | No | Yes | Yes | HCM |
| HCM 14^†^ | DSH | MN | 13 | 3.2 | 3/9 | | 1.76 | 20.3 | 7.5 | 8.3 | 55.6 | Yes | No | No | HCM |
| HCM 15^†^ | DSH | MN | 2 | 4 | - | | 2.94 | 25.5 | 8.5 | 10.8 | 41 | No | Yes | No | HCM |
| HCM 16^†^ | DSH | MN | 2 | 4 | 3/9 | | - | - | - | - | - | - | - | - | HCM |
| HCM 17^†^ | Siamese | MN | 11 | 4.7 | 4/9 | | - | - | - | - | - | - | Yes | - | HCM |
| HCM 18^†^ | DSH | MN | 5.1 | 4.8 | 4/9 | | 2 | 19.3 | 6.8 | 7.2 | 39 | Yes | No | No | HCM |
| HCM 19^†^ | DSH | MN | 3.5 | 2.9 | - | | 2.6 | 22.1 | 4.1 | 4.1 | 16 | No | Yes | No | HCM |
| HCM 20^†^ | BSH | MN | 10 | 5.2 | - | | 3.1 | - | - | - | - | - | Yes | No | HCM |
| HCM 21^†^ | DSH | MN | 7 | - | | - | - | - | - | - | - | - | Yes | No | HCM |
| HCM 22^†^ | BSH | MN | 4 | 3.6 | | - | 1.8 | 21.1 | 3.8 | 8 | 35.4 | No | No | No | HCM |
| HCM 23^†^ | DSH | FN | 8 | 5 | | 4/9 | 2.4 | - | - | - | - | - | - | - | HCM |

Note: Cats (Control 1-10; HCM 1-10) were also used in another publication (doi.org/10.3390/ani13132112). *Cats only used for Western blotting. ‡Cats used for both Western blotting and immunohistochemistry. †Cats used only for immunohistochemistry.

HCM, hypertrophic cardiomyopathy; DSH, domestic shorthair (outbred cats); MN, male neutered; FN, female neutered; BCS, body condition score; La, left atrium; Ao, aorta; IVSd, thickness of interventricular septum in diastole; LVFWd, thickness of left ventricular freewall in diastole; LV FS%, left ventricular fraction shortening (%); SAM, systolic anterior motion; SEC, spontaneous echo contrast.
