# Supplementary material for: Desmin disorganisation: A key feature in feline hypertrophic cardiomyopathy
Source: PLoS One. 2025 Jul 14;20(7):e0327850. doi: 10.1371/journal.pone.0327850 (PMC12258562; doi:10.1371/journal.pone.0327850)
Supplement: S2 Table — (DOCX) [file pone.0327850.s002.docx]

**Table S2** Clinical presentation and echocardiography summary:

| Identifier | Presenting Signs | Murmur | Arrhythmia | Gallop | Co-morbidities | CHF | Reason for euthanasia | Echocardiography summary | Cardiac T-Fast |
| --- | --- | --- | --- | --- | --- | --- | --- | --- | --- |
| Control 1^‡^ | Coughing | Yes | No | No | Solitary pulmonary carcinoma lesion | No | Poor prognosis, Owner request | Normal | - |
| Control 2^‡^ | Lethargy | No | No | No | Hepatic carcinoma | No | Owner request | - | Normal |
| Control 3* | Lethargy, weight loss | No | No | No | IRIS II CKD (controlled SBP), cognitive dysfunction | No | Cognitive dysfunction | Normal | - |
| Control 4^‡^ | Trauma | No | No | No | Road trauma | No | Fractured pelvis and urethral rupture | - | - Normal |
| Control 5^‡^ | Lethargy, weight loss | No | No | No | IRIS II CKD (controlled SBP), Pancreatic neoplasia | No | Abdominal neoplasia | Normal | - |
| Control 6^‡^ | Normal | No | No | No | None | No | Unsociable behaviour | - | - Normal |
| Control 7* | Normal | No | No | No | None | No | Aggression, soiling in house | - | - |
| Control 8^‡^ | Anuria | No | No | No | Toxicity/Acute Kidney Injury | No | Progressive hyperkalaemia | - | Normal |
| Control 9^‡^ | Road Trauma | No | No | No | Road Trauma | No | Multiple trauma | - | - Normal |
| Control 10* | Normal | No | No | No | None | No | Unsociable behaviour | - | - Normal |
| Control 11^†^ | Tachypnoea, pyrexia | No | No | No | Oesophagitis Pyothorax | No | Obtundation | - | Normal |
| Control 12^†^ | - | - | - | - | Concurrent peritonitis, hyperbilirubinemia | No | Biliary disease | - | Normal |
| HCM 1^‡^ | Lethargy, shallow breathing | No | No | Yes | None | Yes | Financial constraints | Mild LAE, LVH, pleural fluid | - |
| HCM 2^‡^ | - Anorexia, weight loss, murmur | - Yes | - No | - No | Hepatic/pancreatic disease “Triaditis” | No | Poor prognosis | - Mild LAE, LVH | - |
| HCM 3* | Hypotension, hypothermia | Yes | No | No | None | Yes | Poor prognosis | LAE, severe LVH, pleural fluid | - |
| HCM 4^‡^ | Lethargy, anorexia, mild tachypnoea | No | No | No | Pulmonary mass | Yes | Poor prognosis | Severe LAE, Pleural fluid | - |
| HCM 5^‡^ | Hind limb paresis and pain | No | No | No | None | Yes | ATE, poor prognosis | LAE, severe LVH, minimal pleural fluid | - |
| HCM 6^‡^ | Hind limb paresis and pain | Yes | Yes | Yes | None | No | ATE, poor prognosis | LAE, LVH, SEC | - |
| HCM 7* | Tachypnoea, inappetence | Yes | No | Yes | None | Yes | Financial constraints | - | LAE, LVH, pleural fluid |
| HCM 8^‡^ | Tachypnoea, stupor, hypotension | No | Yes | No | Diabetes, azotaemia | Yes | Poor prognosis | LAE, LVH, moderate pleural fluid, pulmonary oedema | - |
| HCM 9* | Collapse episode | Yes | Yes | No | None | No | Financial constraints/Owner request | - | LAE, LVH |
| HCM10* | Hind limb paresis and pain | No | Yes | No | None | No | ATE, died in clinic | - | LAE. SEC. LVH |
| HCM 11^†^ | Hind limb paresis/pain | Yes | Yes | No | None | Yes | 2nd ATE episode, poor prognosis | HCM, severe LAE, SEC | - |
| HCM 12^†^ | Hind limb paresis/pain | Yes | No | No | None | Yes | ATE, poor prognosis | - | LVH, LAE with SEC and thrombus |
| HCM 13^†^ | Hind limb paresis/pain tachypnoea | No | No | No | None | Yes | ATE, hyperkalaemia, reperfusion injury | Severe LAE and LVH with CHF and ATE | - |
| HCM 14^†^ | Acute dyspnoea | Yes | No | Yes | None | Yes | Pulmonary oedema | Severe LAE and LVH, CHF | - |
| HCM 15^†^ | Tachypnoea | Yes | No | No | None | Yes | Poor prognosis | Severe LAE, LVH, mid-ventricular obstruction, SEC, pleural fluid | - |
| HCM 16^†^ | Collapsed, tachypnoea | Yes | No | Yes | None | Yes | Cardiogenic shock | - | LVH, LAE, pulmonary oedema (B-lines) |
| HCM 17^†^ | Hind limb paresis and pain |  |  |  |  | No | ATE | - | LAE, LVH, SEC |
| HCM 18^†^ | Hind limb paresis and pain, tachypnoea | Yes | No | No | None | Yes | ATE, Poor response to treatment | LVH, LAE, pulmonary oedema (B-lines) | - |
| HCM 19^†^ | Tachypnoea | No | No | Yes | None | Yes | Poor prognosis, financial constraints | Previous evidence of LVH walls now thinner with evidence of myocardial infarct, LAE, SEC, mild pleural fluid | - |
| HCM 20^†^ | Hind limb paresis and pain, tachypnoea | No | No | No | None | Yes | ATE, poor prognosis | - | Severe LVH, LAE, SEC |
| HCM 21^†^ | Dyspnoea, tachypnoea, hind limb paresis, hypothermia | No | No | No | None | Yes | ATE, poor prognosis | - | LVH, LAE, SEC, scant pericardial fluid, pulmonary oedema (B-lines) |
| HCM 22^†^ | Severe tachypnoea | Yes | Yes | No | None | Yes | Refractory heart failure | LVH, infarcted IVS, pleural fluid, LAE, enlarged RA and RV | - |
| HCM 23^†^ | Dyspnoea and tachypnoea- | - | - | - | - | Yes | Cardiogenic pleural effusion, euthanised as poor prognosis | - | LAE, LVH, pleural effusion |

Note: Cats (Control 1-10; HCM 1-10) were also used in another publication (doi.org/10.3390/ani13132112). *Cats only used for Western blotting. ‡Cats used for both Western blotting and immunohistochemistry. †Cats used only for immunohistochemistry.

HCM, hypertrophic cardiomyopathy; IRIS, international renal interest society; CKD, chronic kidney disease; SBP, systolic blood pressure; CHF, congestive heart failure; T-fast, thoracic focused assessment with sonography for trauma and triage; ATE, aortic thromboembolism; LAE, Left atrial enlargement; LVH, Left ventricular hypertrophy; SEC, spontaneous echo contrast.
