# Supplementary material for: Desmin disorganisation: A key feature in feline hypertrophic cardiomyopathy
Source: PLoS One. 2025 Jul 14;20(7):e0327850. doi: 10.1371/journal.pone.0327850 (PMC12258562; doi:10.1371/journal.pone.0327850)
Supplement: S3 Table — (DOCX) [file pone.0327850.s003.docx]

**Table S3.** Information on echocardiographic views and procedures.

|  | LA/Ao | LVFWd and IVSd | FS (%) |
| --- | --- | --- | --- |
| View | RPSAX at the level of the aortic valve | RPLAX4ch, RPLAX5ch, and RPSAX at the level of the papillary muscles | - |
| Imaging Modality | 2D Imaging | 2D Imaging | - |
| Timing | Beginning of diastole, the first frame of aortic valve closure | End-diastole, the last frame before the aortic valve opens (RPLAX5ch), the first frame after the mitral valve closes (RPLAX4ch), or when the left ventricular internal diameter was the largest (RPSAX) | - |
| Measurement | Ao: From the blood-tissue interface at the midpoint of the right aortic sinus to the commissure between the noncoronary and left coronary aortic cusps LA: Extension of the aortic line to the blood-tissue interface of the left atrial wall, immediately lateral to the pulmonary vein | Leading edge technique avoiding the papillary muscles or false tendon attachments Average of 3 measurements from the area that measures the maximal thickness is used | Calculated using (LVIDd−LVIDs)/LVIDd |

Abbreviations: RPSAX, right parasternal short axis view; RPLAX4ch, right parasternal long axis 4 chamber view; RPLAX5ch: right parasternal long axis 5 chamber view; LVIDd and LVIDs: Left ventricular internal diameter end diastole and end systole; LA: left atrium; Ao: aorta; FS: fraction shortening
