# Supplementary material for: Desmin disorganisation: A key feature in feline hypertrophic cardiomyopathy
Source: PLoS One. 2025 Jul 14;20(7):e0327850. doi: 10.1371/journal.pone.0327850 (PMC12258562; doi:10.1371/journal.pone.0327850)
Supplement: Attachment — Submitted filename: HCM in cats 2025.docx [file pone.0327850.s004.docx]

Hypertrophic cardiomyoathy (HCM) in human and rodents is characterized by an increase in wall thickness and myocyte disarray. HCM is often accompanied by alterations in the intracellular organisation of the intermediate filament system formed by desmin. The authors demonstrate that similar alterations of desmin organisation can also be observed in feline HCM cardiomyocytes. Desmin disorganisation can result from a number of different causes: genetic alterationsm of desmin itself or of desmosome components or an inadequate protein quality control system.

The authors demonstrate that feline hearts affected by HCM show phenotypic changes similar to reported alteration in human or rodent HCM hearts. They identify a disorganised distribution of desmin and the formation of large intra-myocyte aggregates composed of desmin and its chaperone αB-crystalline. Since Western blots indicate an intracellular increase in desmin, it is suggested that the protein quality control system is overloaded.

The disorganisation of desmin is probably causing a disorganization of junctional complexes leading to aberrant localisation of a number of proteins normally residing Z-discs and intercalated discs, such connexin 43, N-cadherin and β-catenin localizing at the lateral sides of HCM cardiomyocytes.

The data are well documented and clearly presented and fully merit acceptance for publication.

Minor points:

In the available version were no Figure legends.

Therefore it was not possible to understand Figure 6, since no information about the meaning of the y-axis was given. In particular, the aim of the procedure used (Cepstrogram) appeared unclear. Was it a particular structure (desmin disorganization and aggregates) of the images to be analysed?

There are some typos.
