# Supplementary material for: Desmin disorganisation: A key feature in feline hypertrophic cardiomyopathy
Source: PLoS One. 2025 Jul 14;20(7):e0327850. doi: 10.1371/journal.pone.0327850 (PMC12258562; doi:10.1371/journal.pone.0327850)
Supplement: Attachment — Submitted filename: Revised Response to Reviewers.docx [file pone.0327850.s005.docx]

PONE-D-25-16588

Desmin disorganisation: a key feature in feline hypertrophic cardiomyopathy

PLOS ONE

Dear Dr. Connolly,

Thank you for submitting your manuscript to PLOS ONE. After careful consideration, we feel that it has merit but does not fully meet PLOS ONE’s publication criteria as it currently stands. Therefore, we invite you to submit a revised version of the manuscript that addresses the points raised during the review process.


Please submit your revised manuscript by Jun 11 2025 11:59PM. If you will need more time than this to complete your revisions, please reply to this message or contact the journal office at [plosone@plos.org](mailto:plosone@plos.org). When you're ready to submit your revision, log on to <https://www.editorialmanager.com/pone/> and select the 'Submissions Needing Revision' folder to locate your manuscript file.

Please include the following items when submitting your revised manuscript:

- A rebuttal letter that responds to each point raised by the academic editor and reviewer(s). You should upload this letter as a separate file labeled 'Response to Reviewers'.
- A marked-up copy of your manuscript that highlights changes made to the original version. You should upload this as a separate file labeled 'Revised Manuscript with Track Changes'.
- An unmarked version of your revised paper without tracked changes. You should upload this as a separate file labeled 'Manuscript'.

If you would like to make changes to your financial disclosure, please include your updated statement in your cover letter. Guidelines for resubmitting your figure files are available below the reviewer comments at the end of this letter.

If applicable, we recommend that you deposit your laboratory protocols in protocols.io to enhance the reproducibility of your results. Protocols.io assigns your protocol its own identifier (DOI) so that it can be cited independently in the future. For instructions see: [https://journals.plos.org/plosone/s/submission-guidelines#loc-laboratory-protocols](https://eur01.safelinks.protection.outlook.com/?url=https%3A%2F%2Ftrack.editorialmanager.com%2FCL0%2Fhttps%3A%252F%252Fjournals.plos.org%252Fplosone%252Fs%252Fsubmission-guidelines%2523loc-laboratory-protocols%2F1%2F010f01967a514f3d-7ebc7bb6-3839-42d2-a912-8dccb7cbe156-000000%2FHaTtUNDDEeGzkj96f46ScIWEXnUWsZeSDMgnouDWVA8%3D208&data=05%7C02%7Cdconnolly%40rvc.ac.uk%7Ca8457f8fb1474df91ace08dd860000a2%7C45a3be7c94024fbead438d1faebfb42d%7C0%7C1%7C638814056793749136%7CUnknown%7CTWFpbGZsb3d8eyJFbXB0eU1hcGkiOnRydWUsIlYiOiIwLjAuMDAwMCIsIlAiOiJXaW4zMiIsIkFOIjoiTWFpbCIsIldUIjoyfQ%3D%3D%7C0%7C%7C%7C&sdata=Cgn6L5TifXJeMlnw6rTUuEIvrsXVsES9cHrNQT31BOU%3D&reserved=0). Additionally, PLOS ONE offers an option for publishing peer-reviewed Lab Protocol articles, which describe protocols hosted on protocols.io. Read more information on sharing protocols at [https://plos.org/protocols?utm_medium=editorial-email&utm_source=authorletters&utm_campaign=protocols](https://eur01.safelinks.protection.outlook.com/?url=https%3A%2F%2Ftrack.editorialmanager.com%2FCL0%2Fhttps%3A%252F%252Fplos.org%252Fprotocols%253Futm_medium%3Deditorial-email%2526utm_source%3Dauthorletters%2526utm_campaign%3Dprotocols%2F1%2F010f01967a514f3d-7ebc7bb6-3839-42d2-a912-8dccb7cbe156-000000%2Fu4v0-wcwbXqeX_pT8vb217XpKESsTEi2PZEpgO_mu2k%3D208&data=05%7C02%7Cdconnolly%40rvc.ac.uk%7Ca8457f8fb1474df91ace08dd860000a2%7C45a3be7c94024fbead438d1faebfb42d%7C0%7C1%7C638814056793777792%7CUnknown%7CTWFpbGZsb3d8eyJFbXB0eU1hcGkiOnRydWUsIlYiOiIwLjAuMDAwMCIsIlAiOiJXaW4zMiIsIkFOIjoiTWFpbCIsIldUIjoyfQ%3D%3D%7C0%7C%7C%7C&sdata=KJLf85f7rIKJjEas5CphXNrs62NhgG9GD0sMiWJl5G0%3D&reserved=0).

We look forward to receiving your revised manuscript.

Kind regards,

Andreas Brodehl, Ph.D.
Academic Editor
PLOS ONE

Journal Requirements:

When submitting your revision, we need you to address these additional requirements.

1. Please ensure that your manuscript meets PLOS ONE's style requirements, including those for file naming. The PLOS ONE style templates can be found at <https://journals.plos.org/plosone/s/file?id=wjVg/PLOSOne_formatting_sample_main_body.pdf> and <https://journals.plos.org/plosone/s/file?id=ba62/PLOSOne_formatting_sample_title_authors_affiliations.pdf>

2. PLOS ONE now requires that authors provide the original uncropped and unadjusted images underlying all blot or gel results reported in a submission’s figures or Supporting Information files. This policy and the journal’s other requirements for blot/gel reporting and figure preparation are described in detail at <https://journals.plos.org/plosone/s/figures#loc-blot-and-gel-reporting-requirements> and <https://journals.plos.org/plosone/s/figures#loc-preparing-figures-from-image-files>. When you submit your revised manuscript, please ensure that your figures adhere fully to these guidelines and provide the original underlying images for all blot or gel data reported in your submission. See the following link for instructions on providing the original image data: <https://journals.plos.org/plosone/s/figures#loc-original-images-for-blots-and-gels>.
In your cover letter, please note whether your blot/gel image data are in Supporting Information or posted at a public data repository, provide the repository URL if relevant, and provide specific details as to which raw blot/gel images, if any, are not available. Email us at [plosone@plos.org](mailto:plosone@plos.org) if you have any questions.

3. To comply with PLOS ONE submissions requirements, in your Methods section, please provide additional information regarding the experiments involving animals and ensure you have included details on (1) methods of sacrifice, (2) methods of anesthesia and/or analgesia, and (3) efforts to alleviate suffering.

4. [Please include captions for your Supporting Information files at the end of your manuscript, and update any in-text citations to match accordingly. Please see our Supporting Information guidelines for more information: http://journals.plos.org/plosone/s/supporting-information.](https://eur01.safelinks.protection.outlook.com/?url=http%3A%2F%2Ftrack.editorialmanager.com%2FCL0%2Fhttp%3A%252F%252Fjournals.plos.org%252Fplosone%252Fs%252Fsupporting-information.%2F1%2F010f01967a514f3d-7ebc7bb6-3839-42d2-a912-8dccb7cbe156-000000%2F48gnMwXhpxLSv-CM80DCAoJesbg9smP5aLnRXMN2hfE%3D208&data=05%7C02%7Cdconnolly%40rvc.ac.uk%7Ca8457f8fb1474df91ace08dd860000a2%7C45a3be7c94024fbead438d1faebfb42d%7C0%7C1%7C638814056793792034%7CUnknown%7CTWFpbGZsb3d8eyJFbXB0eU1hcGkiOnRydWUsIlYiOiIwLjAuMDAwMCIsIlAiOiJXaW4zMiIsIkFOIjoiTWFpbCIsIldUIjoyfQ%3D%3D%7C0%7C%7C%7C&sdata=n8x%2Bz2UfXQNkel3MZ37YUEh0jvyOzm6eDktQhU6tCyk%3D&reserved=0)

5. Please review your reference list to ensure that it is complete and correct. If you have cited papers that have been retracted, please include the rationale for doing so in the manuscript text, or remove these references and replace them with relevant current references. Any changes to the reference list should be mentioned in the rebuttal letter that accompanies your revised manuscript. If you need to cite a retracted article, indicate the article’s retracted status in the References list and also include a citation and full reference for the retraction notice.


[Note: HTML markup is below. Please do not edit.]

Reviewers' comments:

Reviewer's Responses to Questions

**Comments to the Author**

1. Is the manuscript technically sound, and do the data support the conclusions?

The manuscript must describe a technically sound piece of scientific research with data that supports the conclusions. Experiments must have been conducted rigorously, with appropriate controls, replication, and sample sizes. The conclusions must be drawn appropriately based on the data presented.

Reviewer #1: Yes

Reviewer #2: Yes

Reviewer #3: Yes

Reviewer #4: Partly

Reviewer #5: Yes

Reviewer #6: Yes

2. Has the statistical analysis been performed appropriately and rigorously?

Reviewer #1: Yes

Reviewer #2: Yes

Reviewer #3: N/A

Reviewer #4: Yes

Reviewer #5: Yes

Reviewer #6: I Don't Know

3. Have the authors made all data underlying the findings in their manuscript fully available?

The [PLOS Data policy](https://eur01.safelinks.protection.outlook.com/?url=http%3A%2F%2Ftrack.editorialmanager.com%2FCL0%2Fhttp%3A%252F%252Fwww.plosone.org%252Fstatic%252Fpolicies.action%2523sharing%2F1%2F010f01967a514f3d-7ebc7bb6-3839-42d2-a912-8dccb7cbe156-000000%2FQ3TZp7yPw_1OYznFRJ4AIKqhzE0b1PjFrlfLl1sA3cU%3D208&data=05%7C02%7Cdconnolly%40rvc.ac.uk%7Ca8457f8fb1474df91ace08dd860000a2%7C45a3be7c94024fbead438d1faebfb42d%7C0%7C1%7C638814056793805574%7CUnknown%7CTWFpbGZsb3d8eyJFbXB0eU1hcGkiOnRydWUsIlYiOiIwLjAuMDAwMCIsIlAiOiJXaW4zMiIsIkFOIjoiTWFpbCIsIldUIjoyfQ%3D%3D%7C0%7C%7C%7C&sdata=4K0rLdSvmb%2FQV0WGQbiMkNavru7ocHV%2FktaRrgSzz8A%3D&reserved=0) requires authors to make all data underlying the findings described in their manuscript fully available without restriction, with rare exception (please refer to the Data Availability Statement in the manuscript PDF file). The data should be provided as part of the manuscript or its supporting information, or deposited to a public repository. For example, in addition to summary statistics, the data points behind means, medians and variance measures should be available. If there are restrictions on publicly sharing data—e.g. participant privacy or use of data from a third party—those must be specified.

Reviewer #1: No

Reviewer #2: Yes

Reviewer #3: Yes

Reviewer #4: No

Reviewer #5: Yes

Reviewer #6: Yes

4. Is the manuscript presented in an intelligible fashion and written in standard English?

PLOS ONE does not copyedit accepted manuscripts, so the language in submitted articles must be clear, correct, and unambiguous. Any typographical or grammatical errors should be corrected at revision, so please note any specific errors here.

Reviewer #1: Yes

Reviewer #2: Yes

Reviewer #3: Yes

Reviewer #4: Yes

Reviewer #5: Yes

Reviewer #6: Yes

5. Review Comments to the Author

Please use the space provided to explain your answers to the questions above. You may also include additional comments for the author, including concerns about dual publication, research ethics, or publication ethics. (Please upload your review as an attachment if it exceeds 20,000 characters)

## Reviewer #1:

I have reviewed a manuscript by Wan-Ching Cheng et al. investigating changes of desmin and associated proteins in cats with hypertrophic cardiomyopathy.

This is a well written manuscript with a carefully conducted study, a good number of animal samples were used. However, the investigations are somewhat descriptive. I cannot see an immediate benefit to veterinary practice. We thank the reviewer for taking the time to assess our manuscript. We agree that this work is unlikely to result in an immediate benefit to veterinary practice. However, exploring fundamental cellular alterations in cardiomyocytes from HCM affected cats is an important step to help understand potential pathological principles. For instance, desmin disruption may lead to mal location of connexin43 resulting in malignant arrhythmia and sudden cardiac death seen in cats with HCM. Given that PLOS ONE is not an exclusively clinically based Journal we felt this manuscript aligned well with the broader themes of the journal.

A few points should be addressed before publication:

1.) How specific are the changes for Hypertrophic Cardiomyopathy in cats. Could they be a hallmark for any type of heart failure? What is known in the literature? Thank you for this comment, we agree the changes we describe are not unique to HCM in humans and as we pointed out in the introduction (Lines 62-64) have been identified in other cardiac diseases including dilated cardiomyopathy, mitral valve disease and as a result of desmin mutations. That said, the overwhelming cause of feline heart disease is HCM. We have added a sentence to the limitations section to emphasise this point. LINES:486-488 untracked manuscript file “Seventh, it is important to emphasise that the cellular changes described here are not unique to HCM but can occur in other forms of cardiac disease (17,19, 20).”

2.) Introduction goes straight into molecular changes. What are symptoms of HCM (in humans and cats) and how is HCM diagnosed in cats? What is cardiac T-fast? Please explain. In response to you comment we have added a brief paragraph outlining the prevalence and clinical signs in feline HCM as well as diagnostic methods. LINES: 44-58 untracked manuscript file “Hypertrophic cardiomyopathy (HCM) is a serious disease in humans and cats and exhibits considerable similarities at the molecular, cellular and whole organ levels [1-5]. It is characterized by a hypertrophied and non-dilated left ventricle in the absence of abnormal loading conditions capable of producing left ventricular hypertrophy (Luis Fuentes et al 2020). HCM is the most common heart disease in cats affecting up to 15% of the general feline population (Payne JR et al 2015). Echocardiography is the clinical gold standard for HCM diagnosis in cats, and the most used imaging modality in humans (Luis Fuentes et al 2020, Elliott P et al 2014). Clinical signs in cats include respiratory distress due to congestive heart failure, pelvic limb ischaemic paralysis/paresis due to aortic thromboembolism or sudden arrhythmogenic death. Prognosis for cats with HCM is very variable, with median survival times ranging from 596 to 1276 days (Novo Matos J 2023). The role of genetic testing is very limited in cats as to date only two disease associated variants in MYBPC3 have been identified, one in the Maine coon and one in the Ragdoll breed; additionally, a potentially pathogenic variant was found in MYH7 in a domestic shorth air (Raffle J et al 2025).”

We have used the abbreviation T-fast but based on a comment from another reviewer the correct term TFAST should be used. TFAST is a rapid thoracic ultrasound examination used in an emergency situation to evaluate cardiac, pulmonary, mediastinal and pleural structures. This will enable the clinician to identify the presence of obvious cardiac abnormalities such as HCM and atrial thrombi as well as abnormal fluid accumulation such as pleural effusion or pulmonary oedema indicative of congestive heart failure. We have updated our abbreviation throughout and explained the technique in the M+M section. LINES: 107-110 untracked manuscript file “Complete echocardiographic examination or Cardiac TFAST (a rapid thoracic ultrasound examination used in an emergency situation with the animal generally in sternal recumbency optimising the image to evaluate cardiac, pulmonary, mediastinal and pleural structures) was performed in 11/12 control group cats.”

3.) The references to Protein Quality Control are speculative and should be toned down throughout the manuscript in the absence of data on activity of the Ubiquitin Proteasomal System and Autophagy. Thank you for this comment, we agree and have made suitable changes throughout the manuscript.

4.) I assume genetic testing is not done routinely in cats. Could the authors give an overview which HCM disease genes/variants are common in cats of various breeds? You are correct very limited genetic testing is performed in cats as a result of the sparsity of information about disease causing variants. To date two disease associated variants in MYBPC3 have been identified, one in the Maine coon and one in the Ragdoll breed. doi: 10.1016/j.ygeno.2007.04.007, doi: 10.1093/hmg/ddi386. Additionally, a potentially pathogenic variant was found in MYH7 in a domestic shorthair. We have added a sentence to the introduction to this effect LINES: 53-58 untracked manuscript file “The role of genetic testing is very limited in cats as to date only two disease associated variants in MYBPC3 have been identified, one in the Maine coon and one in the Ragdoll breed; additionally, a potentially pathogenic variant was found in MYH7 in a domestic shorth air (Raffle J et al 2025).”

Would it make sense to identify pure breeds (likely to be inbred) and outbred cats int table S1/S2? In table S1 we identify which cats are outbred and which are purebred. All cats designated DHS (domestic shorthair) are outbred, we have added a sentence to the table legend to clarify this.

5.) Figures 1 and 13 should also feature desmosomes. Thank you for this comment, the figures have been updated in line with your request.

6.) Check immunofluorescence figures for sufficient resolution (e.g. Figs 2 and 5 look blurry) Thank you for the comment - We agree that on the submitted build PDF the figures do look blurry as shown by the indistinct labelling. This blurriness is not present on our actual figures and therefore likely is an issue with the PDF building programme – I will defer to the Editor.

7.) Figure 3 (Western blots) why are only few samples used here, but much higher n numbers in Figure 10? We thank the reviewer for this helpful comment. The difference in sample numbers reflects the sequential development of the study. Desmin was initially analysed in 5 HCM and 5 control cats, which was the pre-defined scope of the original investigation focused on intermediate filament disruption. Upon observing striking findings in desmin expression, we decided to further examine its chaperone protein αB-crystallin in the same group of animals to assess the relationship between their expression levels.

Later, we sought to explore junctional proteins and analysed β-catenin using sample resources from a parallel study on myocardial fibrosis (reference 37). This set included five additional cats per group, resulting in a total of 10 HCM and 10 control cats, and enabled an expanded analysis of β-catenin expression.

We have clarified these experimental phases and sample numbers in the revised Materials and Methods section LINES: “Five control and 5 HCM cats were used for immunoblotting of desmin and αB-crystallin; and 10 control and 10 HCM cats were used for junctional protein β-catenin immunoblotting. αB-crystallin was analysed in the same set of cats as used for desmin to assess paired expression. β-catenin was analysed using membrane resources from a parallel study on myocardial fibrosis (reference 37), which included five additional cats in each group.”

Show full blots with all samples in Suppl. Material. for all western blots. Our apologies if these full blots were not available to you – again they were uploaded when we build the PDF – I will re-upload and defer to the Editor if they do not appear again

Figure 3B swap alpha-beta crystalline (to go on top) with Gapdh. Thank you for your helpful suggestion. We have updated Figure 3B to reflect the correct arrangement, with αB-crystallin now shown on top and GAPDH (loading control) below, as per standard western blot presentation format.

8.) Figure 6: label y-axis, show individual data points per animal? We have amended the figure as requested.

9.) Figure 12: label what is shown in green and red Thank you for the comment in our legends we clarify which protein is labeled red and which green. For figure 8: Desmin – red; β-catenin – green; Nuclei – blue. For figure 12: Desmin – red; Connexin43 – green; Nuclei – blue

## Reviewer #2:

The manuscript titled "Desmin disorganisation: a key feature in feline hypertrophic cardiomyopathy" presents compelling evidence that intermediate filament (IF) disruption, particularly the aggregation of desmin and αB-crystallin, constitutes a significant pathological feature of feline hypertrophic cardiomyopathy (HCM). The authors provide robust histological and biochemical data, supported by cepstrogram analysis and immunostaining, which align the features of feline HCM with established human pathomechanisms. The paper is generally well-structured, and the findings possess considerable translational relevance. We thank the reviewer for their supportive words

However, several critical comparisons (e.g., desmin, αB-crystallin, connexin43) are based on limited and inconsistent sample sizes across groups. The authors should provide a rationale for the allocation of samples across assays and to clarify the measures taken to ensure statistical robustness. We thank the reviewer for this valuable comment. The discrepancies in sample numbers across assays were due to a combination of practical factors inherent to working with clinical samples rather than standardized laboratory models. Our initial experimental design targeted 5 HCM and 5 control cats for each analysis. However, due to tissue limitations (e.g., exhausted paraffin blocks), loss of slides during shipping, occasional failed staining, and limited availability of some antibody reagents, we supplemented with additional cases when possible.

Furthermore, control hearts are inherently more difficult to obtain compared to clinical HCM cases, especially when histologically confirmed as normal. This contributed to some differences in group sizes.

We acknowledge that our initial table included incorrect n values (Table 1), which have now been corrected. Despite these variations, all statistical comparisons (when performed) were performed using appropriate non-parametric tests suited for small and unbalanced groups based on discussions with our College statistician.

In addition to cepstrogram analysis and qualitative immunostaining, have the authors quantified desmin disorganization (e.g., through fluorescence intensity or spatial metrics)? If such quantification has been conducted, it should be included in the manuscript; if not, the authors should consider whether such quantification would enhance the interpretation of their data. Thank you for this insightful comment. Our cepstral analysis was derived as a way of determining the desmin organization as this is key to one of the central themes of the paper that desmin disorganisation impacts other important adhesion proteins etc. We did not quantify the degree of desmin disorganisation, but this is something that could be considered in future studies.

The Western blot results indicate an accumulation of desmin and αB-crystallin. Did the authors assess ubiquitin tagging or proteasome function to substantiate claims of protein quality control dysfunction? It would be beneficial to evaluate proteasome markers. Thank you for this comment we agree that this would have been very interesting to perform but was beyond the scope of the PhD which forms the basis of this work. Additionally in line with a similar comment from Reviewer 1 we have altered the discussion and added to the limitations section to reflect that this study has not confirmed that dysfunction of protein quality control was the cause of the changes to desmin quantification we identified and other possible explanations such as increased synthesis was not ruled out in this study.

The discussion addresses potential mitochondrial dysfunction related to IF disruption. Have the authors considered employing transmission electron microscopy (TEM) or immunostaining for mitochondrial proteins such as VDAC or TOM20 in these samples? We did not perform this analysis but again we thank the reviewer for these excellent suggestions which hopefully will form the basis of future work in this exciting area.

Were any of the HCM-affected cats genotyped (e.g., for the MYBPC3-A31P mutation in Maine Coons)? Genetic stratification could elucidate the inter-individual variability observed in desmin pathology and connexin43 dislocation. Genotyping was not performed in any of the cats including the two Maine Coons – please refer to our response to Reviewer 1 regarding the status of genotyping in diagnosis of feline HCM. Specifically regarding the MYBPC3-A31P mutation - the picture even within the Maine coon breed is mixed with both genotype +ve/phenotype -ve and genotype -ve/phenotype +ve individuals (J Vet Cardio. 2010 Dec;12(3):155-61. doi: 10.1016/j.jvc.2010.06.004)

While the abnormal localization of connexin43 suggests electrical uncoupling, the authors should consider incorporating electrophysiological assessments (e.g., ECG or optical mapping) to establish a connection between these findings and arrhythmic risk. The study would significantly benefit from functional readouts, such as calcium handling, contractility, or conduction velocity, particularly to support the mechanistic link to sudden cardiac death. Again, we thank the reviewer for these excellent suggestions. Unfortunately, ECG data was not available for most of the HCM cats as they invariably presented to our out of hours emergency service. However, none experienced sudden cardiac death and rather were euthanised for the reasons outlined in Table S2. Arrhythmias were detected by auscultation in 6/23 cats but these were intermittent and not considered haemodynamically significant. ECGs were performed in two cats that were subsequently transferred to the Cardiology service (HCM 11 and HCM 22) in both cats, intermittent uniform ventricular premature complexes were identified. Regarding optical mapping this is not a technique we have available in our institution and given the cardiac tissue used in this study came from clinical cases (not laboratory animals) the logistics associated with collaborating with another institution to perform OM would be hugely challenging.

## Reviewer #3:

Critical Evaluation of ‘Desmin disorganization: a key feature in feline hypertrophic cardiomyopathy’

The article presents an analysis of cytoskeletal disruption, mostly desmin disorganization and its impact of chaperone and structural proteins in cardiomyocytes in feline hypertrophic cardiomyopathy tissues.

Although, the co-localization of desmin and its chaperone (αB-crystallin) and ID proteins such as β-catenin and connexin43 were discussed and studied, it remains unclear whether desmins disruption drive the misslocation of the mentioned proteins. Mechanistic experiments such as knockdown or rescue studies would have reinforce the connection between desmin, αB-crystallin, β-catenin and connexin43. We thank the reviewer for their input into our manuscript. We agree with the Reviewer and accept that this is primarily a descriptive study that will hopefully lead to further investigation. The further studies you suggest were beyond the scope of this initial study however, we have added to the discussion to indicate that functional studies are required to confirm the link between desmin disorganisation and the other proteins studies. LINES: 443-445 untracked manuscript file “However, without mechanistic experiments such as knockdown or rescue studies we cannot say for certain if the miss-location of these important junctional proteins is a consequence of desmin disruption.” We have made a similar reply to other reviewers.

Furthermore, due to the results of western blot experiments, the quantitative level of the proteins were discussed without having transcriptional data to rule out increased synthesis. We agree with this point and have added a comment to that effect in the discussion and limitations section. Unfortunately, logistically given that most of the cats were admitted to our overnight emergency service (with limited staffing) it was difficult to process the tissue for both RNA and protein analysis. LINES: 403-409 untracked manuscript file “Furthermore, we show increased quantities of desmin and αB-crystallin in the HCM cats by western blot which likely reflect overload of the PQC system and failure to remove the accumulated aggregates (Fig 3), however we did not perform transcriptional analysis on these samples to ascertain increased synthesis as an alternative explanation for increased protein levels. Furthermore, proteasome function tests were not evaluated in this study to directly quantify proteasome activity and protein quality control function.”

Next, the sex bias of investigated cats were mentioned (p=0.07) but without further discussion. Thank you for this comment, we were restricted to use the feline clinical cases that presented to the hospital over the time of the study. It is not surprising that the HCM group had a male bias as the increased prevalence of the disease is well recognised in the feline population (J Vet Cardiol.2015 Dec:17 Suppl 1:S244-57.doi:10.1016/j.jvc.2015.03.008). We have added a sentence to this effect in the discussion. LINES: 463-464 untracked manuscript file “The increased prevalence of male cats in the HCM group compared to control was not surprising as a male sex predisposition is well recognised in the feline population (Payne JR et al 2015).”

A genotype analysis was not performed for a further study of genetic factors of HCM. Please see our response to the other reviewers concerning the status of genotyping in the feline population.

Despite these limitations, the article contribute a feline perspective on HCM research.

## Reviewer #4:

In the manuscript the authors describe that in feline HCM- similiar to human HCM- aggregates of Desmin an alphaB-crystalline may occur . In addition they also observed a disorganization of components of adherens and gap junctions, which suggest an impaiered cell-cell communication. Since there is not much known about it in cats, this is an important study , though there are some majors concerns, listed below. We thank the reviewer for their help with improving this manuscript.
I appreciated that in the discussion sections the limitations of this study are described. I But I also would have preferred some information on the cats specifities, in case of anomalous findings. Thank you for this comment, we are unsure what extra information the reviewer requires beyond the detailed information given in Supplementary Tables 1 and 2 which include cat details (age/sex/breed etc), presenting signs, reason for euthanasia, echocardiography or TFAST results and pathology details. We would be happy to assist the reviewer but need more information on what they require.

I had problems to access Fig. 13 Only a fast glimpse was possible and I did not find any full blot images in the supplements as was announced. We are sorry that you could not access these files (another reviewer mentioned a similar problem). The files were all uploaded onto the Journal Website when the PDF was being built - and I can see them in my copy. I will defer to the Editor regarding this issue.

Major concerns:
1) Line 51 ff. What is meant by „comparable“ the statement a completely refer to human CM. Thank you for this comment – We agree this is rather ambiguous and have changed the sentence accordingly in the manuscript and reordered the paragraph to distinguish between human and feline studies. LINES: 64-68 untracked manuscript file “The development of such aggregates is suggestive of consistent with IF disorganisation and inadequate protein quality control (PQC) which underlies the cells' inability to completely remove the toxic accumulation of malformed proteins and suggests that IF disruption represents a shared cellular response to a variety of genetic and acquired cardiac insults.

What about feline CM. Is there know anything know about IF dysfunctuntion/disorganization in cats. There is no literature available regarding feline CM and alteration in IF organisation - we have added a sentence to the introduction to make this clearer. LINES: 68-73 untracked manuscript file “There is a sparsity of information describing IF configuration in feline cardiomyocytes or the impact of HCM on their spatial arrangement. However, in one study cytoplasmic aggregates of unknown composition were detected in cardiomyocytes from cats with HCM and in a second study a variable pattern of staining for desmin was identified in HCM cats compared to controls but cytoplasmic aggregates were not seen [2, 30].”

Please distinguish and describe clearly the situation in cats vs human. Thank you for this comment, we have clarified this in the manuscript and explain that very little is known in cats other than what we referenced
2) Fig.1 it would be grat, if you could also mark costameres, desmosomes and th and gap/ adherens junctions in the summary. Not all readers are familiar with this. In general give at much information as possible in the figures as they should be self-explanatory. Thank you for this comment - we have updated the figure in line with your request.

3) Line 86ff. This paragraph is not very clear. It sound as if ECGs and cardiac TFASts have been performed with dead cats. Cardiac T-fast to my knowledge is an expression for tachycardia and cardiac TFAST an ultra sonic examination method, which should be explained shortly (e.g.alternating left and right lateral recumbency / sternal recumbency, image optimization) We apologise for this lack of clarity and have adopted the correct term TFAST and give an explanation of how it was conducted in the M+M section. LINES: 107-110 untracked manuscript file “Complete echocardiographic examination or Cardiac TFAST (a rapid thoracic ultrasound examination used in an emergency situation with the animal generally in sternal recumbency optimising the image to evaluate cardiac, pulmonary, mediastinal and pleural structures) was performed in 11/12 control group cats.”

4) Line 99ff why ony some cats were selected exclusively for western blotting and others only for immunofisochemistry, why not all for both? Do controls and hcm slected tissue match in age, gender. Thank you for this comment – the reason for some cats being used for WB some for IHC and some for both depended on the quantity of material available and whether the overnight staff had time to dissect the heart and remove a section of the left ventricular free wall (lateral wall) and freeze for WB analysis. Please see our reply to Reviewer 3 for a further explanation. Regarding age the selected tissue do match, however given the increased prevalence of HCM in male cats, more tissue from male cats will be present in the HCM group compared to control.

5) Lines211ff does it make sense to calculate a median age, since the range is very large. Wouldn’t it be better to select 2 different age groups e.g ≤7 and >7? We have added a sentence to the results section describing the % of cats > 7 years in both groups. LINES: 245-247 untracked manuscript file “As the age range was large, we also assessed the percentage of cats >7 or ≤ 7 years in both groups. In the control group 41.7% (5/12 cats) were > 7 years, in the HCM group 47.8% (11/23 cats) were > 7 years.”

6) Isn‘t it possible to combine some figures into one e.g. Fig 3 and 4 For an easier understanding Thank you for your helpful suggestion. As recommended, we have combined Figure 3 and Figure 4 into a single composite figure (now presented as Figure 3 in the revised manuscript)

please mark the axis of fig 6 e.g. with degree of disorder or something similar This has been done as requested.
7) Line 275 f which oft he cats investigated had a severe disruption of desmin architecture: are thes cats older? Are male or female? Specific race, differences in HCM progression etc? should be discussed.

We appreciate the reviewer’s thoughtful question. Please see below the table listing the 6 cats used for the analysis of desmin and beta-catenin immunostaining.

| PlosOne Supp | Desmin, beta-catenin | Breed | Gender | Age |
| --- | --- | --- | --- | --- |
| HCM 14 | Des some at ID; b-C most still at ID | DSH | MN | 13 |
| HCM 16 | Des overall weak and not at ID; b-C some at ID | DSH | MN | 2 |
| HCM 17 | Des not at ID; b-C still at ID | Siamese | MN | 11 |
| HCM 18 | Des some at ID; b-C most still at ID | DSH | MN | 5.1 |
| HCM 19 | Des not at ID and mildly weak; b-C not at ID | DSH | MN | 3.5 |
| HCM 21 | Des not at ID and very disorganised; b-C not at ID | DSH | MN | 7 |

The cats with the most severe desmin disorganisation were cases HCM 21 and HCM 16. Both were domestic shorthair (DSH) neutered males aged 7 and 2 years, respectively. Although these cats share sex and breed, their ages differ significantly, and our limited sample size does not allow us to draw strong conclusions regarding age or breed associations. However, both cats exhibited advanced clinical signs (supplementary table S2), including congestive heart failure, thromboembolism, or cardiogenic shock, suggesting that severe desmin disorganisation may reflect disease severity or progression rather than demographic factors.

8) Legend to Fig 9 concerning the 3 cats where no lateralization was observed see comment 7
We thank the reviewer for pointing this out. As described in the manuscript (Lines 283–286), we assessed N-cadherin localisation in 3 HCM cats, as both N-cadherin and beta-catenin are components of the adherens junction. In these 3 HCM cats, N-cadherin was no longer clearly localised to the intercalated discs, and instead appeared more diffusely distributed within the cytoplasm, contrasting with the well-defined ID localisation seen in control cats.

Given the small number of cases examined, we are currently unable to determine whether N-cadherin consistently undergoes lateralisation in feline HCM. However, we can conclude that its localisation in these HCM cats differs from the control pattern, with loss from the intercalated discs.

9) Line 424 which post-translational modifications you are thinking of ( oxidative modifications, phosphprylation) Thank you for this comment we agree there are a number of possible PMT including one pointed out by another reviewer concerning the cleavage of desmin as a potential PMT linked to aggregate formation and or mislocalization (DOI: 10.1002/humu.20941; doi:10.1093/cvr/cvu003; doi: 10.1096/fj.07-088724).”

10) Which consequences do the results described here could have for treatment/management of feline HCM what are the future aspects? Immediate future aspects we aim to undertake would be analysis of the proteosome in control and HCM cats so we can be more confident in our hypothesis that the desmin disorganisation and aggregate formation described in this paper are a direct result of dysfunction of the protein quality control system. Regarding treatment/management of feline HCM we feel that this work is at a very early stage and unlikely to lead to immediate treatment options in the short term. Certainly, if IF disorganisation does result in mitochondrial dysfunction in HCM cats then this would be a potential therapeutic pathway to explore going forward.

Minor comments Thank you for pointing out these typing errors we have corrected them as requested.
1) A few typing mistakes: Line 76: … ß-catenin, and intracellular…; line 104: desribed; line 244: ,respectively. We have corrected these
2) Supplement table 2: in the legend FS is defined, which is not found in the table only FN or F Thank you for pointing this out – the abbreviation has been changed appropriately
3) Line 74 please define freewallk ( part of ventricle wall not connected to septum or apex?) Thank you for this comment – your description is correct we will add this to the manuscript for clarity
4) Shouldn’t We are uncertain what you are pointing out here?
5) Line 288: …. Expression of ß-catenine observed by ?immunostaining. We have changed this as requested
6) Line 179 figure heading shouldn’t cepstrogram analysis be replaced by cepstral analysis since cepstrogram ist he result of cepstarl analysis? Thank you yes we agree and have changed the figure heading as requested.
7) Line 292 heading fig.10 : expression cannot be increased by immuno blotting – Thank you for this – yes very true we have improved the wording

## Reviewer #5:

Hypertrophic cardiomyoathy (HCM) in human and rodents is characterized by an increase in wall thickness and myocyte disarray. HCM is often accompanied by alterations in the intracellular organisation of the intermediate filament system formed by desmin. The authors demonstrate that similar alterations of desmin organisation can also be observed in feline HCM cardiomyocytes. Desmin disorganisation can result from a number of different causes: genetic alterationsm of desmin itself or of desmosome components or an inadequate protein quality control system.
The authors demonstrate that feline hearts affected by HCM show phenotypic changes similar to reported alteration in human or rodent HCM hearts. They identify a disorganised distribution of desmin and the formation of large intra-myocyte aggregates composed of desmin and its chaperone αB-crystalline. Since Western blots indicate an intracellular increase in desmin, it is suggested that the protein quality control system is overloaded.
The disorganisation of desmin is probably causing a disorganization of junctional complexes leading to aberrant localisation of a number of proteins normally residing Z-discs and intercalated discs, such connexin 43, N-cadherin and β-catenin localizing at the lateral sides of HCM cardiomyocytes.
The data are well documented and clearly presented and fully merit acceptance for publication. We thank the reviewer for their kind and encouraging words.

Minor points:
In the available version were no Figure legends. We are sorry that the figure legends were not visible to you. They were uploaded to the Journal when the PDF was built. Hopefully they will be visible when I re-upload otherwise I will defer to the editor.
Therefore it was not possible to understand Figure 6, since no information about the meaning of the y-axis was given. Thank you – appropriate labelling of the y axis has been done. In particular, the aim of the procedure used (Cepstrogram) appeared unclear. Was it a particular structure (desmin disorganization and aggregates) of the images to be analysed? The approach basically attempts to quantify 'parallelness' of sarcomeric staining using cepstral analysis. In the case of any increase in disorder, the cepstral components become less defined and the normal distribution of angular components becomes broader. We have modified the manuscript to make the aim of performing this analysis clearer. LINE: 288-290 untracked manuscript file “Given the disorganisation of desmin in the HCM cats identified by immunoblotting we performed two-dimensional cepstral analysis on LV sections from (7 control cats) and (16 HCM cats) as a way of further determining the desmin organization in cardiomyocytes from diseased cats (Fig 5).” And LINE: 400-403 untracked manuscript file “Additionally, our cepstral analysis supports and quantifies the disorganisation of the IF network in HCM cats identified by immunostaining which is important since a key aim of our study was to determine the impact of IF disorganisation on other key associated proteins (Fig 56).”

There are some typos. We will address these - thank you for bringing them to our attention.

## Reviewer #6:

Based in published data that cytoplasmic aggregates of the intermediate filament desmin and its chaperone heat shock protein αB-crystallin together with disorganisation of associated junctional proteins including β-catenin, N-cadherin and connexin43 are identified in cardiomyocytes from human HCM patients and mouse models in the present study Wan-Ching Cheng and et al. sought to identify and further characterise the localisation of these proteins in a cohort of cats with HCM. They used a pool of 12 control and 23 HCM feline cardiac samples for their analysis.
By immunostaining they showed similar aggregate formation and/or mislocalization of these proteins in feline HCM cardiomyocytes and by western blot increased expression of desmin, αB-crystallin and N-cadherin. Their data are interesting and are suggestive of common subcellular responses of cardiomyocytes to HCM in mammals. We would like to thank the reviewer for scrutinising our manuscript and for their helpful suggestions.

I have some comments for the presented data:

• First, I think the group of the HCM animals lacks information. As indicated for the control samples (used in another publication (doi.org/10.3390/ani13132112), representative histopathological features of HCM feline hearts (HE, Masson's Trichrome…) should be presented. It will be informative to have the same area by histological staining next to the immunofluorescence stained samples.

We thank the reviewer for this thoughtful comment and agree that including conventional histological images alongside immunostained sections can enhance interpretation. In our previous publication (doi:10.3390/ani13132112), we used chromogenic immunostaining, which allowed better visualisation of tissue architecture and alignment with H&E or Masson’s Trichrome stained serial sections.

In contrast, the present study employed immunofluorescence, which by nature results in visually sparse or transparent sections under brightfield, making it technically very difficult to identify or relocate the exact anatomical region for subsequent routine histological staining. At the time of study design and imaging, the substantial challenge of spatial correlation between fluorescence-labelled sections and histological landmarks meant that such paired visualisation was not pursued.

We emphasise that all HCM cases were histologically confirmed by specialist veterinary pathologists (MD, LW) based on H&E and Masson’s Trichrome staining, following established diagnostic criteria. We have MT images of HCM cat 11 and HCM cat 13 (Tables S1 and S2) showing cardiomyocyte disarray and interstitial fibrosis and in the case of cat 13 replacement fibrosis as well. We are happy to add these as a separate figure to the manuscript maybe as an addition to supplementary materials as combining the MT staining with one of the IHC figures in the manuscript would not make sense in our opinion as we cannot guarantee that the sections match. Would the reviewer be happy with this?

HCM Cat 13 (1mm)	HCM Cat 13 (500µm)

HCM Cat 11 (500µm)

• Is genetic analysis for HCM samples available or already published? (doi.org/10.1038/s41598-025-87852-5). It will be nice to be included. Thank you for this comment which has been raised by a number of reviewers. Thank you for this comment, genotyping was not performed on these cats as explained in our reply to Reviewers 1, 2, and 3.
• Figure 2 (legend) says: “Confocal micrographs of feline hearts” As indicated in methods “Image analysis. Fluorescent labelling of the proteins was detected using a Leica DMRA2 upright microscope. Is not confocal microscope and I think for this type of analysis confocal microscope analysis is needed as photos are blurry!

We thank the reviewer for this observation. The use of the term “confocal” in the figure legend was an oversight and has been corrected to “microphotograph” to accurately reflect the imaging method. All immunofluorescence images were acquired using a Leica DMRA2 upright widefield microscope.

Although confocal microscopy provides superior Z-axis resolution, our paraffin-embedded sections were cut at 4 μm thickness, which helps minimize out-of-focus signal and improves axial resolution compared to standard 6–8 μm sections. This enhances the reliability of spatial signal interpretation, even without confocal optics.

To objectively support our qualitative interpretation of overlapping signal patterns between desmin and αB-crystallin, we performed quantitative image analysis on representative high-resolution TIFF images using the Coloc 2 plugin in Fiji (ImageJ). The results confirmed strong spatial correlation:

• Figure 5C (longitudinal section):

Pearson’s R = 0.74, Manders’ tM1 = 0.920, tM2 = 0.911, Costes P = 1.0

• Figure 5C (transverse section):

Pearson’s R = 0.78, Manders’ tM1 = 0.853, tM2 = 0.817, Costes P = 1.0

These results validate our initial visual interpretation and demonstrate that the observed overlap between desmin and αB-crystallin is statistically significant and unlikely to result from random signal distribution.

We acknowledge that the previously submitted images may have appeared blurred due to unintentional downsampling during figure preparation. To address this, we have reconstructed the figure using the original high-resolution TIFF files. The revised figure now reflects the full image quality (see revised Figures 2 and 5).

For the reagent control sample a control of HCM sample should be also included.

We thank the reviewer for raising this point. The reagent controls shown for both fluorescence channels are from HCM cats and we have updated our figure with extra labelling indicating the tissues are from HCM cats.

• Some figures I think could be combined as for example figure 1 and 13, figure 2 and 5, figure 3 and 4, figures 11 and 12 Thank you for this comment it was also mentioned by a previous reviewer, we agree and we have combined figures where we feel it is appropriate

• As the authors mention in the limitation section, post-translational modifications (PMT) of the proteins analysed would give a more in depth understanding to how these proteins are regulated. Regarding desmin, cleavage of desmin could be a PMT linked to aggregate formation and or mislocalization (DOI: 10.1002/humu.20941; doi:10.1093/cvr/cvu003; doi: 10.1096/fj.07-088724). Over exposure of desmin western blot (figure 3) could be informative.

We appreciate the reviewer’s thoughtful comment and agree that desmin cleavage may contribute to protein mislocalisation and aggregation. The Western blot protocol used in this study was optimised for full-length desmin detection under conditions that minimise background. Additional exposures were not performed as part of the original analysis. We agree this is a valuable direction for future investigation.

• As a lot of the HCM animals had Hind limb paresis it will be nice to include a desmin immunostaining of skeletal muscle tissue section. We thank the reviewer for this interesting comment. Unfortunately, we only had ethical approval to take cardiac tissues from these cats so obtaining skeletal muscle sections was not possible.

6. PLOS authors have the option to publish the peer review history of their article ([what does this mean?](https://eur01.safelinks.protection.outlook.com/?url=https%3A%2F%2Ftrack.editorialmanager.com%2FCL0%2Fhttps%3A%252F%252Fjournals.plos.org%252Fplosone%252Fs%252Feditorial-and-peer-review-process%2523loc-peer-review-history%2F1%2F010f01967a514f3d-7ebc7bb6-3839-42d2-a912-8dccb7cbe156-000000%2FjsjgGm6ZRJlNa-wMo2m9mtJdCFwmWbgfPsizDiJrU5s%3D208&data=05%7C02%7Cdconnolly%40rvc.ac.uk%7Ca8457f8fb1474df91ace08dd860000a2%7C45a3be7c94024fbead438d1faebfb42d%7C0%7C1%7C638814056793818396%7CUnknown%7CTWFpbGZsb3d8eyJFbXB0eU1hcGkiOnRydWUsIlYiOiIwLjAuMDAwMCIsIlAiOiJXaW4zMiIsIkFOIjoiTWFpbCIsIldUIjoyfQ%3D%3D%7C0%7C%7C%7C&sdata=c9X75pnf3lpb8dF46LS1h%2B4wZh81K7bZFqHOICLP%2FfI%3D&reserved=0)). If published, this will include your full peer review and any attached files.


If you choose “no”, your identity will remain anonymous but your review may still be made public.


**Do you want your identity to be public for this peer review?** For information about this choice, including consent withdrawal, please see our [Privacy Policy](https://eur01.safelinks.protection.outlook.com/?url=https%3A%2F%2Ftrack.editorialmanager.com%2FCL0%2Fhttps%3A%252F%252Fwww.plos.org%252Fprivacy-policy%2F1%2F010f01967a514f3d-7ebc7bb6-3839-42d2-a912-8dccb7cbe156-000000%2FlNyVLI_p42FA4f4aPC1825JCG48tIKgVzUUVNfyRqWQ%3D208&data=05%7C02%7Cdconnolly%40rvc.ac.uk%7Ca8457f8fb1474df91ace08dd860000a2%7C45a3be7c94024fbead438d1faebfb42d%7C0%7C1%7C638814056793831119%7CUnknown%7CTWFpbGZsb3d8eyJFbXB0eU1hcGkiOnRydWUsIlYiOiIwLjAuMDAwMCIsIlAiOiJXaW4zMiIsIkFOIjoiTWFpbCIsIldUIjoyfQ%3D%3D%7C0%7C%7C%7C&sdata=Cb7Cvy9odkuOxp450MSF2nabdlRPCd2%2BTNuk0%2BXzanE%3D&reserved=0).

Reviewer #1: No

Reviewer #2: **Yes:** Nazha Hamdani

Reviewer #3: No

Reviewer #4: No

Reviewer #5: No

Reviewer #6: No

[NOTE: If reviewer comments were submitted as an attachment file, they will be attached to this email and accessible via the submission site. Please log into your account, locate the manuscript record, and check for the action link "View Attachments". If this link does not appear, there are no attachment files.]

While revising your submission, please upload your figure files to the Preflight Analysis and Conversion Engine (PACE) digital diagnostic tool, [https://pacev2.apexcovantage.com/](https://eur01.safelinks.protection.outlook.com/?url=https%3A%2F%2Ftrack.editorialmanager.com%2FCL0%2Fhttps%3A%252F%252Fpacev2.apexcovantage.com%252F%2F1%2F010f01967a514f3d-7ebc7bb6-3839-42d2-a912-8dccb7cbe156-000000%2F6psvY0OrMxwa83FVIC8cW1VNQ9Se7W2NfWk-LNnZ7MM%3D208&data=05%7C02%7Cdconnolly%40rvc.ac.uk%7Ca8457f8fb1474df91ace08dd860000a2%7C45a3be7c94024fbead438d1faebfb42d%7C0%7C1%7C638814056793843595%7CUnknown%7CTWFpbGZsb3d8eyJFbXB0eU1hcGkiOnRydWUsIlYiOiIwLjAuMDAwMCIsIlAiOiJXaW4zMiIsIkFOIjoiTWFpbCIsIldUIjoyfQ%3D%3D%7C0%7C%7C%7C&sdata=I%2Fm6xfv0sJT4taQTBpySv31wT%2BOf3vTgps8nXR6t%2BSk%3D&reserved=0). PACE helps ensure that figures meet PLOS requirements. To use PACE, you must first register as a user. Registration is free. Then, login and navigate to the UPLOAD tab, where you will find detailed instructions on how to use the tool. If you encounter any issues or have any questions when using PACE, please email PLOS at [figures@plos.org](mailto:figures@plos.org). Please note that Supporting Information files do not need this step.
